# Usability of digital medicine systems in patients with schizophrenia or schizoaffective disorder

**DOI:** 10.3389/fpsyt.2026.1903689

**Published:** 2026-10-08

**Authors:** Taishiro Kishimoto, Harumasa Takano, Ryo Kanzaka, Hisateru Tachimori, Hideki Oi, Hiroyuki Ogino, Kyosuke Sawada, Fumiaki Kumagai, Masayuki Tomita, Iku Nakayama, Yoshiyuki Shibasaki, Toshiaki Kikuchi, Hiroyoshi Takeuchi, Kazuyuki Nakagome

**Affiliations:** 1 Center for the Promotion of Interdisciplinary Research in Medicine and life Science, Keio University School of Medicine and Graduate School of Medicine, Tokyo, Japan; 2 Department of Clinical Neuroimaging, Integrative Brain Imaging Center, National Center of Neurology and Psychiatry, Tokyo, Japan; 3 Department of Clinical Data Science, Clinical Research & Education Promotion Division, National Center of Neurology and Psychiatry Hospital, Tokyo, Japan; 4 Department of Healthcare Management, Kyorin University School of Medicine, Tokyo, Japan; 5 Department of Psychiatry, Inokashira Hospital, Tokyo, Japan; 6 Department of Psychiatry, Asaka Hospital, Fukushima, Japan; 7 Department of Psychiatry, Sakuragaoka Kinen Hospital, Tokyo, Japan; 8 Department of Psychiatry, Oizumi Hospital, Tokyo, Japan; 9 Digital Business Office, Otsuka Pharmaceutical Co., Ltd., Tokyo, Japan; 10 Medical Affairs, Otsuka Pharmaceutical Co., Ltd., Tokyo, Japan; 11 Department of Neuropsychiatry, Keio University School of Medicine, Tokyo, Japan; 12 Department of Psychiatry, Chiba University Graduate School of Medicine, Chiba, Japan; 13 Department of Psychiatry, National Center Hospital, National Center of Neurology and Psychiatry, Tokyo, Japan

**Keywords:** digital health, remote patient monitoring, schyzophrenia, treatment adherence, user-centered design

## Abstract

**Objective:**

To assess the usability and performance of digital medication management systems (DMS) for patients with stable ambulatory schizophrenia and schizoaffective disorder.

**Methods:**

This prospective, interventional study comprised a screening phase (up to 4 weeks), training phase (3 weeks: system use under the support), and independent phase (5 weeks under the minimized support) with a follow-up period of 2 weeks. Based on the DSM-5 criteria, patients with a diagnosis of schizophrenia or schizoaffective disorder, taking an atypical antipsychotic as their primary medication, and having access to a smartphone were included in this study.

**Results:**

A total of 30 patients participated in the study. The main characteristics of the patients were as follows: age, 43.5 ± 11.4 years; male percentage, 40% (n = 12); duration of antipsychotic drug use, 14.2 ± 11.7 years; educational period, 13.3 ± 2.1 years; chlorpromazine (CP) equivalent dose, 369.6 ± 253.6 mg/day; and Brief Psychiatric Rating Scale (BPRS), 23.6 ± 4.8. The patients applied patches with the sensor of the DMS embedded in (hereinafter, a wearable sensors) for 93.2% of the study period. The patients could apply the patch independently and successfully and synchronize it with a smartphone application that allowed them to monitor their medication intake in 15.2 ± 8.1 days (median, 9.0; interquartile range (IQR), 1.0–15.0 days after initiation). Thirty-eight adverse events that were reported in 17 patients were classified as mild or moderate, and were related to wearable sensor use.

**Conclusion:**

It was concluded that most patients completed the task for the DMS with minimal assistance early in the study and continued to apply wearable sensors throughout the study period. Moreover, the results confirmed the feasibility of DMS in clinical practical use in stable schizophrenia.

## 1 Introduction

The continuation of antipsychotic medication for relapse prevention has important clinical implications (1, 2), because multiple relapses of psychotic symptoms lead to a decline in mental and social functions in patients with schizophrenia (3). However, poor medication adherence in patients with schizophrenia has not yet been further investigated, despite being a main factor for relapse (4, 5). The methods for confirming medication include self-report, evaluation from surroundings, evaluation by therapists, pill counting, evaluation by prescription, and medication event monitoring System (MEMS) that measures the opening and closing of bottles with an electronic chip. However, medication confirmation in daily practice is still mainly evaluated by a doctor or self-reported by a patient. In general, adherence to a physician’s assessment or patient’s self-report is often overestimated when compared with objective measurement, which has been reported in cases of schizophrenia and bipolar disorder (6–8).

The conventional methods for medication review other than pill count and MEMSs are still insufficient to achieve an accurate assessment of medication adherence. If a method that can more objectively and easily monitor the medication status and allows not only patients but also medical professionals and caregivers to check the medication status on time is available, it can be a useful treatment tool for schizophrenia. Moreover, updated information regarding medication status can be used by medical professionals for medication management in each patient, while the complexity associated with medication confirmation will be reduced for patients, caregivers, and supporters.

Recently, new methods using DMS have been introduced (9). A digital medicine system includes a drug–device combination product comprising oral antipsychotic tablets embedded with an ingestible event marker sensor, wearable sensor, smartphone application to display information for the patient, and web-based portals for healthcare providers and caregivers that display a summary of tablet ingestion over time.

Digital health feedback devices have already been approved in the United States, Europe, and China. Even though the usability of this device has been authorized overseas, it is necessary to confirm whether it can be applicable to schizophrenia patients in Japan, which has different cultures, customs, and medical treatment systems, and whether it has the potential to be a truly effective device. Based on this background, we decided to conduct an exploratory study to assess the usability of DMS in Japanese patients with schizophrenia.

## 2 Methods

### 2.1 Study design

This exploratory prospective and interventional study was conducted in accordance with the Clinical Research Act (Act No. 16 of 2017), with the utmost consideration given to the dignity and human rights of individual research participants and the protection of personal information. Moreover, this study was reviewed and approved by a certified clinical research review committee based on the Clinical Research Act. Before the start of patient enrollment, this study was registered with the Japan Registry of Clinical Trials (jRCTs032190089) https://jrct.mhlw.go.jp/. Before the screening test, the research participants were provided with an explanatory document and consent document approved by an accredited clinical research review committee, and written informed consent of the research participants was obtained. The clinical research coordinator assisted with the written explanations as necessary.

This study consisted of a screening, training, independent, and follow-up phase. In the screening phase (maximum of four weeks), the baseline assessments and system descriptions were provided to eligible participants on the first day of the study. In the training phase (3 weeks (Day 1–22)), the following were necessary for the application of DMS to patients at each site: installation of application software, account registration by using the handle name, method of synchronizing the patch with the application software of the smart phone, proper way of pressing the button of the patch, method of using the application of the smart phone, and method of sticking the patch to the skin of the appropriate position of the body. Moreover, the patients started system registration, device synchronization, and the miniature ingestible event maker in tablet (MIT) medication. Caregivers/supporters or healthcare professionals were able to provide support when using the system as needed. On the second day, the research participants continued to use the system either on their own or with the support of their caregivers. At Week 1 (Day 8), Week 2 (Day 15), and Week 3 (Day 22), the patient’s ability to use the system was assessed. At weekly intervals during the study, medical professionals have assessed whether the participants synchronized a new patch with the application and applied it to the appropriate position on their torso. In the independent phase (five weeks (Day 23–57)), from Week 4 to Week 8, we investigated whether the patients could use the system without any support from a healthcare professional. Furthermore, in the follow-up phase, additional information on adverse events was confirmed during Day 71–73, two weeks after the visit at Week 8.

### 2.2 Patients

The key inclusion criteria were as follows: 18–65-year-old patients diagnosed with schizophrenia or schizoaffective disorder based on the DSM-5 who were taking an atypical antipsychotic (aripiprazole, brexpiprazole, olanzapine, risperidone, or blonanserin) as the base agent in the outpatient setting and who were able to use the smartphone provided by the present study or a smartphone for personal use.

Furthermore, this study excluded substance-dependent patients with intellectual developmental delay, intellectual developmental disorder, dementia, epilepsy, or convulsion. Moreover, patients with other dermatoses, such as wart, exanthema, atopic dermatitis, and skin irritation, were also excluded.

### 2.3 Medication and DMS

During the study, the patients were administered with atypical antipsychotic tablets and MIT-filled DBcaps capsules^®^, which were the main agents at the time of obtaining consent. If multiple tablets with different strengths of the active ingredient were taken at one time, one tablet was filled into a swallowable capsule. Moreover, the atypical antipsychotic doses were in the range not exceeding the upper limit of the prescribing information. This DMS comprises MIT (chip containing a placebo tablet), patch (wearable sensor), software (Proteus MDDS), application (application for patients), cloud server, and web portal (**Figures 1**, **2**). The ingestible event marker (IEM) of the placebo tablet is activated in the stomach and sends a signal to the Proteus patch, which receives a signal from the IEM and sends a low-power signal to certain devices (smart phone, tablet PC) connected by Bluetooth. The Proteus software in the mobile device receives information from the patch and simultaneously shares information with the application installed in the same device and transmits the data to a cloud server through a portable telephone line and Wi-Fi. The cloud server averages the patient’s medication status and analyzes the trend, whereas the web portal (portal site for healthcare professionals) displays the information analyzed by the cloud server using charts and tables. The patients can refer to their own data on their smartphones, and medical professionals and caregivers can also check the above information on the cloud server.

**Figure 1:**
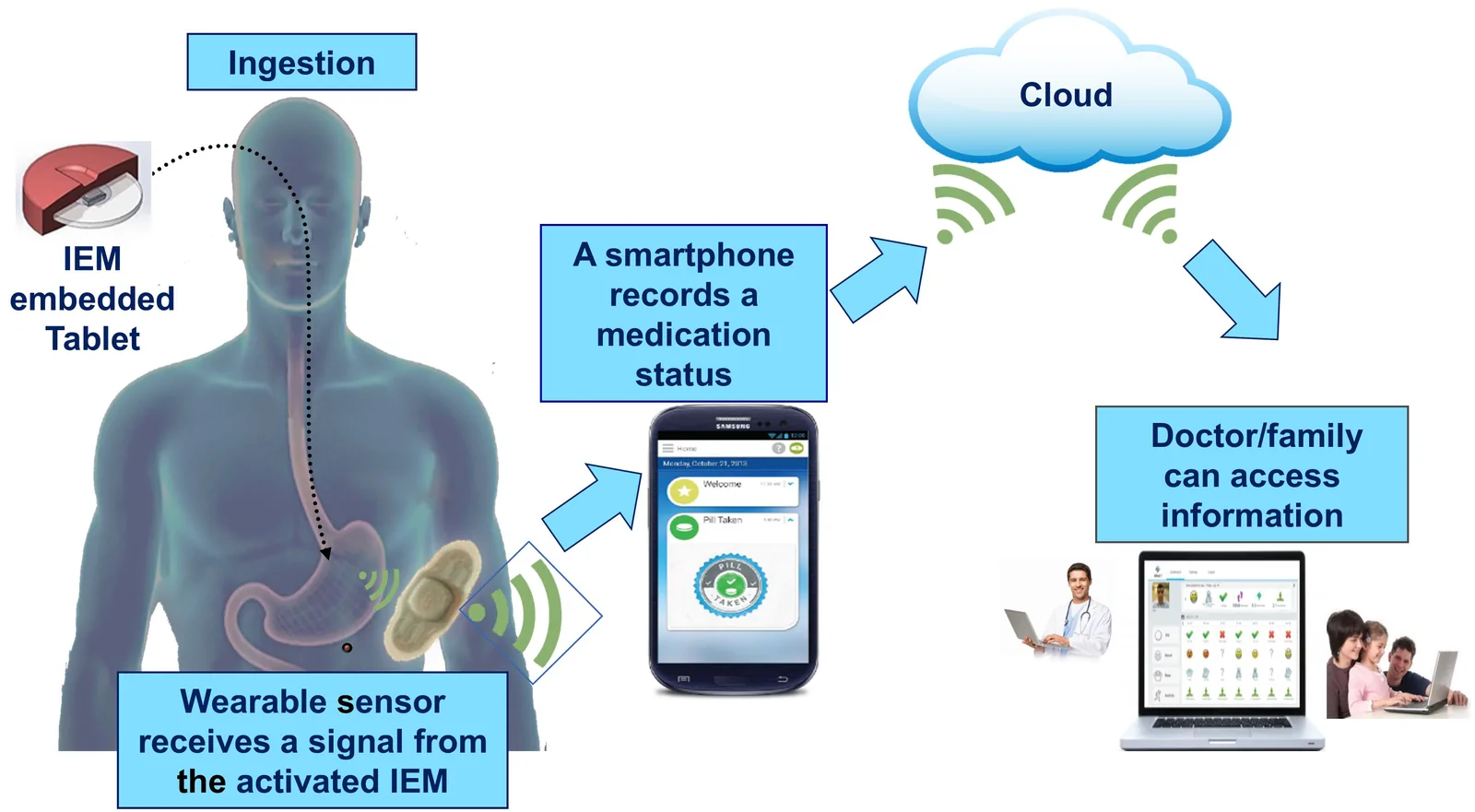
Schematic diagram of a digital medications system. The IEM embedded tablet was filled in the DB®cap capsule with one tablet of antipsychotic. IEM, ingestible event maker.

**Figure 2:**
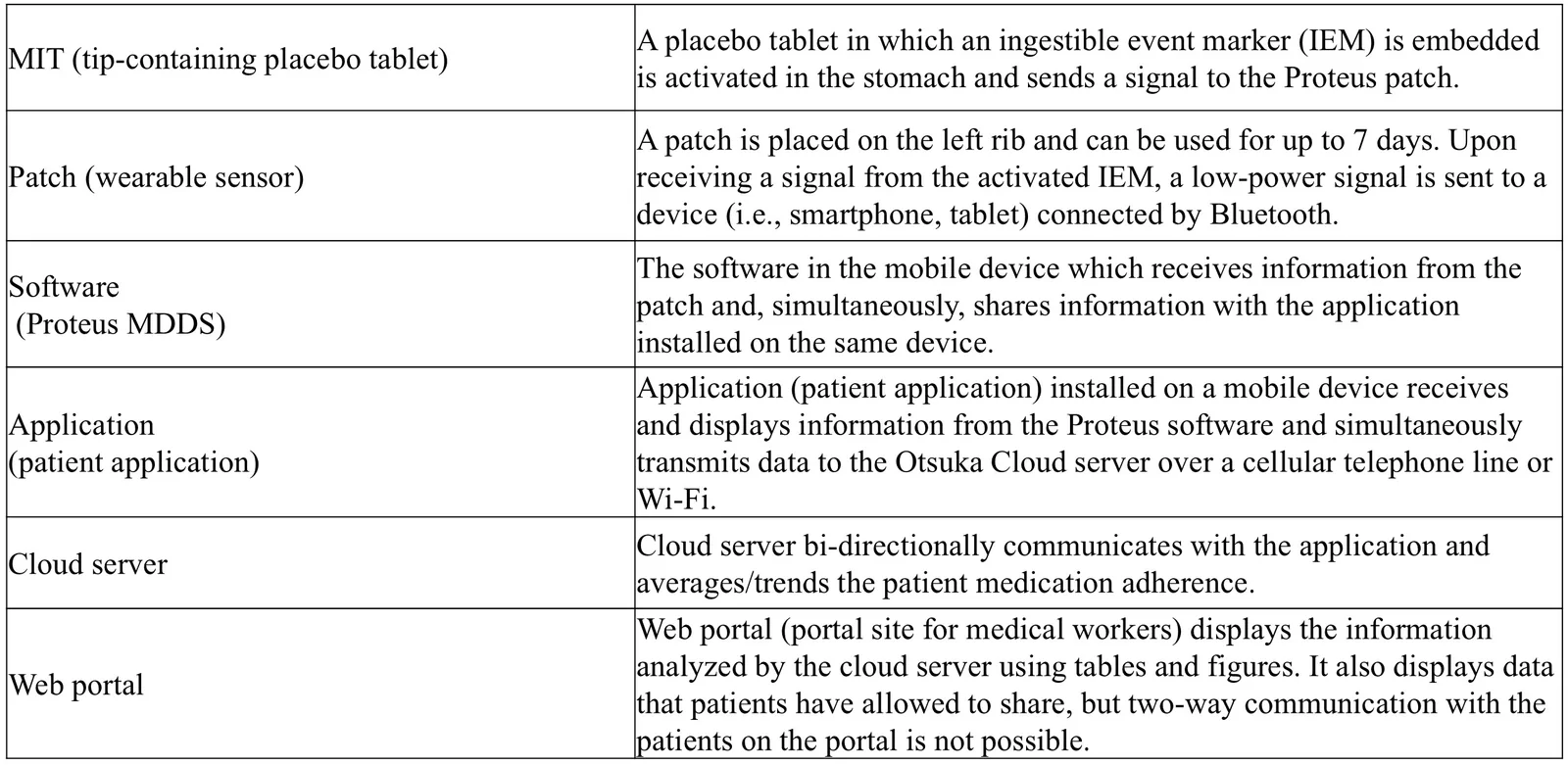
DMS components and the process of data exchange on the website. DMS, digital medication management system. MIT, the miniature ingestible event maker in tablet.

### 2.4 Study outcome measure

The patch wearing time, defined as the duration during which a patient could apply the patch to an appropriate area and synchronize it with the smartphone application, was considered an indicator of proper use of the DMS. Accordingly, the primary outcome measure was set as the proportion of the patch wearing time that the participants wore their patches during the whole research period. The secondary outcome measures were the (a) proportion of the participants who were able to apply the patch independently and successfully and synchronize it with the application; (b) proportion of time when the participants applied the patch independently and successfully and synchronized it with the application (weekly or in each study period); (c) time to the first success of the participant being able to apply the patch independently and successfully and synchronize it with the application; and (d) number of MITs ingested by the participant and number of ingested MITs registered on the digital health data server. The mean change from baseline to Week 8 (or early termination) in Clinical Global Impressions-Severity of Illness Scale (CGI-S) and BPRS and adverse events related to the device and medications were also assessed.

### 2.5 Sample size

The sample size for this study was determined from the number of cases that were considered feasible. In a multicenter, 8-week clinical trial conducted in the U.S (9)., the discontinuation rate was approximately 30%. Accordingly, in the present study, it is estimated that at least 21 study participants would complete the 8-week evaluation period, while 30 participants were planned to be enrolled in the present study.

### 2.6 Statistical analysis

The continuous variables are expressed as mean, standard deviation, median, and quartile range (IQR), whereas the categorical variables are expressed as number and percentage. The adverse events were coded using MedDRA PT. The imputation of missing values was not performed. The analysis software used were SAS 9.4 or higher (SAS Institute Japan, Inc), JMP 13.0 or higher (SAS Institute Japan, Inc), and Microsoft Office Excel 2007 or higher (Microsoft Corp). The primary endpoint (the percentage of appropriate patch wearing) was calculated by dividing the sum of the appropriate patch wearing time observed on the digital health server by the sum of the study participation hours for each study participant. The periods during which the patch was peeled off less than 3 hours were not included in the calculation of the sum of the patch wearing time. Moreover, a sensitivity analysis was performed by changing the definition of patch non-wear time from less than 3 hours to less than 20 minutes.

## 3 Results

### 3.1 General characteristics of the patients

**Figure 3** shows the flow chart of the patients evaluated and included in the study. A total of 31 patients signed informed consent between September 2020 and June 2021, of whom 30 moved to treatment. One patient discontinued the study because the findings suggested clinical worsening, while 29 participants completed the 8-week study. **Table 1** shows the patient demographics and characteristics. The mean age was 43.5 ± 11.4 years, and the male accounted for 40% of the total population (n = 18). The duration of the disease was 16.6 ± 11.4 years, whereas the duration of the antipsychotic medication was 14.2 ± 11.7 years. Moreover, 26.7% (8/30) of the patients were living with family members. 97% (29/30) of patients had an educational history of more than 10 years. The CP equivalent dose was 369.6 ± 253.6 mg/day. At baseline, the Global Assessment of Functioning (GAF), CGI-S, and BPRS were 67.8 ± 14.1, 2.2 ± 1.1, and 23.6 ± 4.8, respectively. Nine patients were administered with aripiprazole, 10 brexpiprazole, 7 olanzapine, 1 risperidone, and 3 blonanserin. The medication compliance rate for the past 4 weeks from the baseline date was 98.0 ± 6.6% as reported by the patients and 97.0 ± 8.5% in the objective assessment by the physician.

**Figure 3:**
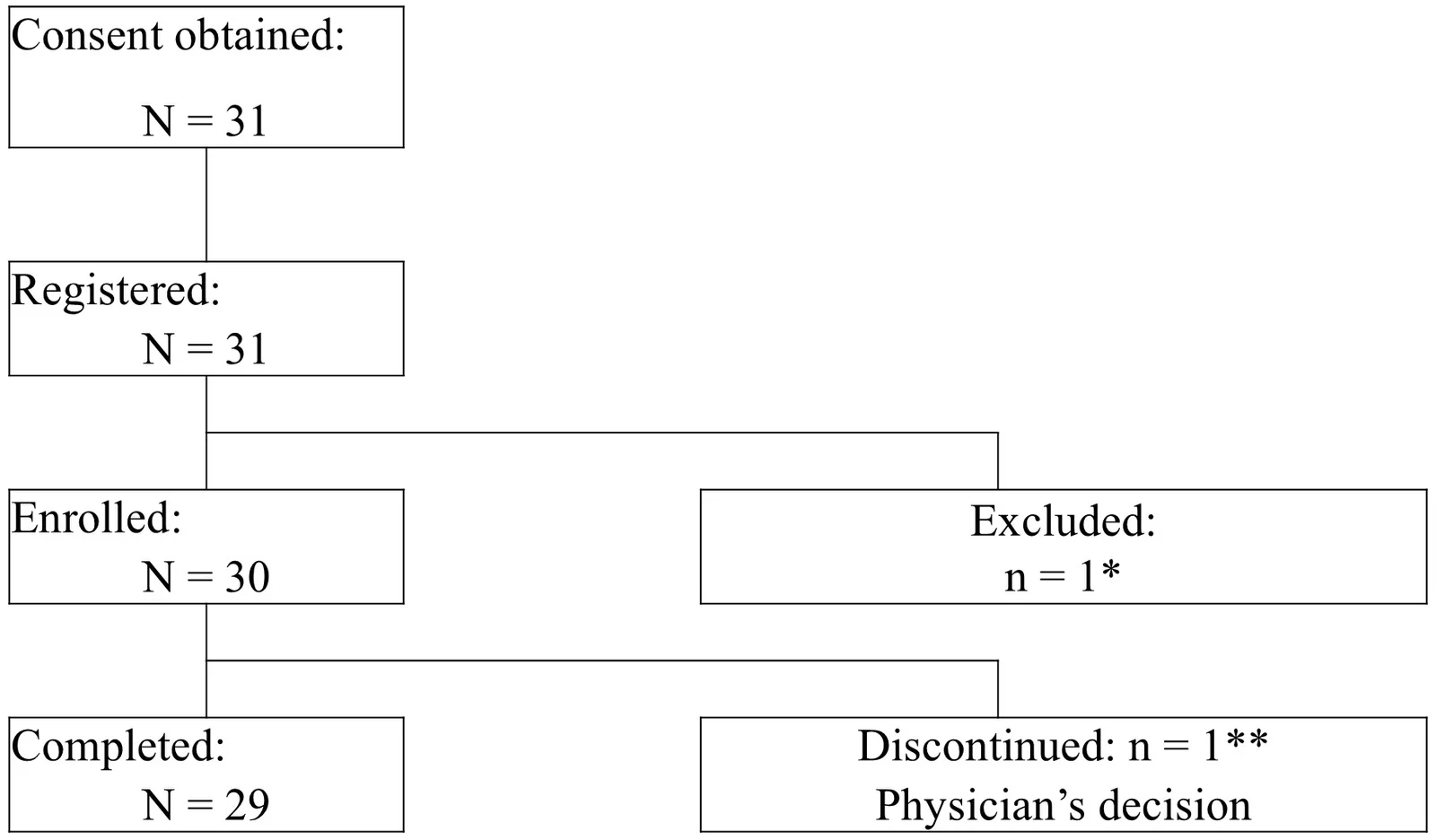
Patient flow chart. *Due to priority given to treatment for diabetes. **Withdrawn at Week 8 due to excessive mental and physical burden.

**Table 1:** Patients’ democratics and characteristics.

Variables	n = 30	Variables	n = 30
Sex		Diagnosis, n (%)	
Male	12 (40.0)	Schizophrenia	27 (90.0)
Female	18 (60.0)	Schizoaffective disorder	3 (10.0)
Age, years	43.5 ± 11.4	Chlorpromazine equivalent dose, mg/day, n (%)	
		< 200	8 (26.6)
BMI, kg/m2	25.3 ± 4.6	≥ 200 to < 400	10 (33.3)
		≥ 400 to < 600	5 (16.6)
Duration of disease, years	16.6 ± 11.4	≥ 600 to < 800	4 (13.3)
		≥ 800 to < 1000	2 (6.6)
Antipsychotic treatment, years	14.2 ± 11.7	1000 <	1 (3.3)
		(mean ± SD)	369.6 ± 253.6
Cohabitation, n (%)			
Yes	8 (26.7)	Number of antipsychotics n (%)	
No	22 (73.3)	1	22 (73.3)
		2	8 (26.7)
Academic history, n (%)			
Junior high school	1 (3.3)	GAF score	67.8 ± 14.1
High school	14* (46.7)		
University	5** (16.7)	CGI score	2.2 ± 1.1
Graduate school	3 (10.0)	0: not assessed	2 (6.7)
Junior colleague	3 (10.0)	1: normal, not at all ill	7 (23.3)
Vocational school	4 (13.3)	2: borderline mentally ill	8 (26.7)
		3: mildly ill	10 (33.3)
Education period, years, n (%)		4: moderately ill	3 (10.0)
< 10	1 (3.3)		
10 to < 15	24 (80.0)	BPRS	23.6 ± 4.8
15 ≤	5 (16.7)	DAI-10 score	6.5 ± 3.6

*Dropout 1. **Dropout 2 and currently attending 1.

Categorical data was indicated by n (%).

Continuous variables are expressed as mean ± standard deviation.

BMI, body mass index; GAF, global assessment of functioning; CGI, Clinical Global Impressions-Severity of Illness; BPRS, Brief Psychiatric Rating Scale; DAI-10, Drug Attitude Inventory-10 Questionnaire.

### 3.2 Primary and secondary outcomes

The proportion of time during which the patients appropriately wore a patch in the entire period was 93.2% (**Table 2**). The subgroup analysis revealed no differences in patch coverage by gender (male, female), age (below median, above median), duration of disease (below median, above median), or educational history (college degree or higher, below college degree), all of which showed values above 90% (**Table 2**). A sensitivity analysis was performed by replacing the definition of patch non-wearing time for less than 3 h to less than 20 min. The proportion of patch wearing time was 91.6%, which was consistent with the result of the primary analysis (93.2%) (**Table 2**).

**Table 2:** Proportion of time patients applied the patches during the whole research period (primary, subgroup, and sensitivity analysis).

Variables	Subgroup	n	% of patch wearing time^#^
Primary analysis (all)*		30	93.2
gender	male	12	96.7
	female	18	90.9
age (years)**	<44.5	15	90.8
	≥44.5	15	95.7
duration of disease (years)**	<14.9	15	91.2
	≥14.9	15	95.3
withdrawal from study	no	29	93.3
	yes	1	88.1
educational history 1	college graduate	5	95.2
	others	25	92.8
educational history 2	high school graduate	28	93.1
	others	2	94.7
Sensitivity analysis (all)***		30	91.6

*Intermittent patch unwearing < 3 hours was not considered as temporary interruption.

**Stratified by each median of age and duration of disease.

***Intermittent patch unwearing < 20 min was not considered as temporary interruption.

#The sum of the time that the study subjects applied patches appropriately during the whole study period on the server was divided by the sum of the length of the time between the first applying the patch and the last wearing off the patch for the subjects.

**Table 3** shows the percentage of patch wearing time by week. The wearing rate was maintained above 90% in most weeks, 92.9% in the training phase, and 93.4% in the independent phase, indicating that the system was appropriately used from the beginning of use.

**Table 3:** Proportion of time patients could apply the patches in training and independent phases.

				Training phase				Independent phase					
Variables		Whole period	(Week)					(Week)					
			Pre	1	2	3	Total	4	5	6	7	8	Total
n		30	30	28	30	30	30	30	29^*^	29^*^	29^*^	30	30
% time of patch wearing**		(93.2)		(96.4)	(88.5)	(93.4)	(92.9)	(91.7)	(92.9)	(93.8)	(95.7)	(92.9)	(93.4)

*One patient skipped the site visit.

**Calculated by dividing the sum of the patch wearing time on the server by the corresponding sum of the study time for each study subject.

**Table 4** shows the percentage of patients who could properly apply the patch without any assistance and synchronize it with the application. The patients who were able to manage this system without any help increased over time (33.3% at Week 1, 42.9% at Week 2, 79.3% at Week 3, and 96.7% at Week 8). It was observed that most patients were found to be able to patch and synchronize it with the applications appropriately on their own during the training period. The mean time to the first occurrence of successful independent patch application and synchronization with the application was 15.2 ± 8.1 days (median, 9.0; IQR, 1.0–15.0). Meanwhile, one patient needed assistance throughout the study.

**Table 4:** Patients who succeeded in applying the patch independently and synchronizing it with the application.

Subgroup		Training phase				Independent phase	Whole period
	Pre	Week 1	Week 2	Week 3	Sub-total	Week 8	
(N)	30	28	30	29	sss30	30	30
Succeeded	10	12	23	23	25	29	29
(%)	(33.3)	(42.9)	(79.3)	(79.3)	(83.3)	(96.7)	(96.7)
Failed	20	16	6	6	5	1	1
(%)	(66.7)	(57.1)	(20.7)	(20.7)	(16.7)	(3.3)	(3.3)

The patient’s ability how completely he/she managed the tasks were assessed by their health care providers at their site visit.

**Figure 4** shows the changes in the self-reported medication rates, application rates on digital health servers, and medication rates on digital health servers. The average rates of self-reported medication, medication on the digital health server, and the patch wearing time on the digital health server during the entire study period were 99.1 ± 2.4%, 89.9 ± 9.0%, and 96.8 ± 6.0%, respectively. No significant difference was observed in the ingestion rate between patient self-reports and digital health server record.

**Figure 4:**
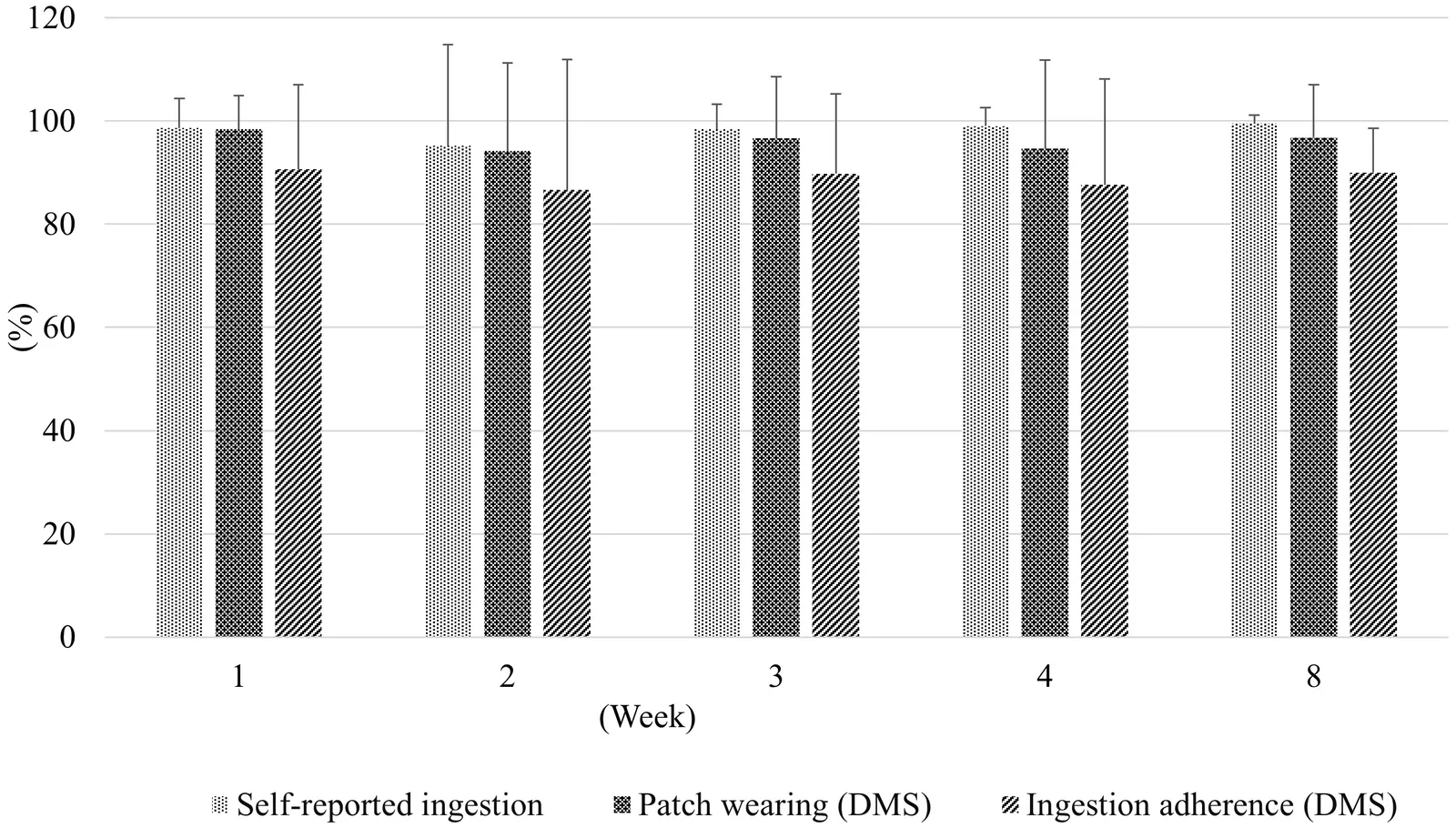
Self-report ingestion rate, ingestion rate on the digital health server, and rate of wearing time on the digital health server. Self-reported ingestion; the ratio of the number of self-reported ingested capsules by the study subjects to the number of capsules prescribed during the relevant period. Patch-wearing time on the server: the ratio of the total time the research subject applied a patch to the total time of the corresponding period. Ingestion rate on the server: the ratio of the number of capsules recorded on the digital health server to the number of prescription (capsules) during the relevant period. Data are expressed as mean ± standard deviation. DMS, digital medication management system.

The CGI-S, BPRS, and Drug Attitude Inventory-10 Questionnaire (DAI-10) at Week 8 were 2.0 ± 0.9, 23.1 ± 4.6, and 6.5 ± 3.8, respectively, and did not differ from the values at baseline.

### 3.3 Safety

A total of 38 adverse events occurred in 17 patients, and the major adverse events were pruritus, erythema, contact dermatitis, and rash, which can be attributed to patch wearing. Of the 38 events, 36 were mild and 2 (pruritus, erythema) were moderate. These moderate adverse events recovered within 2 months. No increase in the adverse events from the training phase to the monotherapy phase was observed (22 vs 8 events).

## 4 Discussion

### 4.1 Key findings and interpretation

The continuation of antipsychotic medication is important to prevent relapse, but poor adherence to medication and disengagement from treatment among schizophrenic patients is an important therapeutic issue that has been insufficiently addressed in Japan. One possible solution to poor medication adherence is the use of long-acting injection (LAI). Kishimoto et al. reported that LAI may be more effective against recurrence than oral agents (10). However, in Japan, the shift to LAI has not progressed as much as in other countries in which LAI is prescribed in approximately 20-30% of patients with schizophrenia (11, 12), with a tendency patients/physicians prefer oral medication. In addition, Onitsuka et al. investigated the usage of psychotropic drugs in Japanese schizophrenia patients, and reported that monotherapy rate of LAI remained at about 40%, and oral antipsychotics were added on LAIs in many patients (12). Given this medical situation in Japan, the implementation of this study to determine how DMS can be useful for Japanese patients with schizophrenia and which patients are better suited to this type of DMS is helpful in solving adherence issues.

The previous study by Peters-Strickland et al. (9) that confirmed the usability of this DMS reported the baseline characteristics of the patients as follows: mean age, 46.6 years; gender, 74.6% male; disease duration, 19.3 years and CGI-S (borderline ill, 4.5%; mildly ill, 70.1%; moderately ill, 22.4%; and markedly ill, 3.0%). On the other hand, in the present study, the mean age of the study participants was 43.5 years, male percentage 40.0%, disease duration 16.6 years, mean CP equivalent 369.6 mg, and CGI-S 23.3% (borderline ill, 26.7%; mildly ill, 33.3%; and moderately ill, 10%). In 2011, the Psychiatric Clinical Pharmacology Group investigated the prescribing status of 21,823 inpatients with schizophrenia at 149 psychiatric hospitals in Japan. The results indicated a mean age of 58.2 years, male percentage of 51.8%, mean CP equivalent of 816 mg, and monotherapy rate of 34.7% (13). Moreover, Hashimoto et al. (14) investigated the prescription patterns in 2,177 schizophrenia patients in Japan at the time of discharge from 83 facilities (37 university hospitals, 22 national and public hospitals, and 24 private hospitals) and reported a mean age of 46 years, monotherapy rate of 53.7%, and mean CP equivalent of 683 mg. Accordingly, the patient population in this study was considered clinically milder than those surveyed for the actual clinical use of antipsychotics for the treatment of schizophrenia in Japan as well as those in the previous study.

The previous study (9) also reported patient disposition, in which 73.1% (49/67) of the patients completed the study and 26.9% (18/67) of the patients dropped out of the study; the details of which were six due to discontinuation, six due to consent withdrawal, six due to adverse events, four due to lost to follow-up, one due to protocol deviation, and one case of physician’s decision. They also reported an average patch wearing rate of 70.7% during the study period. In contrast, the present study has a completion rate of 96.7% (29/30) and only one case of discontinuation by physician’s decision and showed a patch application rate of 93.2% during the study period. The high percentage both in the completion rate and the patch application rate during the study period might be due to the fact that patients with relatively mild symptoms were included in this study compared with their study. Moreover, the patch wearing rates by gender, age, duration of disease, and educational history did not differ, with a minimum wearing rate of 90% and a maximum wearing rate of 96.7%. These results suggest that appropriate and continuous patch application is highly possible regardless of gender and age, duration of illness, and educational history among patients with moderate or mild symptoms.

In our study, 79.3% of the patients were able to apply the patch and synchronize with the application without any assistance at the end of the training period (at Week 3) and 96.7% at the end of the study (at Week 8), with an average duration of 15.2 days in which the patient successfully managed the DMS. These results suggested that patients become able to handle independently and quickly the DMS, and it is expected that the burden on caregivers and healthcare workers in this system implementation will be limited. In addition, Zhao et al. (15) reported that adherence drastically declines just after patients with schizophrenia are discharged from the hospital. As mentioned above, this DMS can be managed early with minimal assistance. Therefore, the implementation of this system prior to hospital discharge might be practically useful for maintaining adherence. Moreover, in reducing the complication for medication administration during the limited outpatient visit time, it is expected that medical professionals can use their resources more efficiently and this DMS could provide patients, caregivers, and supporters with more accurate information on daily medication intake. In addition, the accurate monitoring of adherence by this DMS could contribute to the decision-making concerning treatment strategy.

The medication adherence rate of 89.1% on the digital health server was consistent with the self-reported medication rate of 99.1%, although some patients might have ingested a tablet when they did not wear a wearable sensor. In general, a large discrepancy was observed between the rate of medication reported by the patients to the healthcare professionals and the actual rate of medication (6, 16). The study data obtained from our study, however, showed higher values than the previous study, which confirmed the usability of this system previously (9) (59.4% of the medication adherence rate on the digital health server). Moreover, it is known that the adherence rate tends to decline as the treatment period increases (15). In contrast to the previous study, the medication rate was maintained in a period of 8 weeks, probably because the patients enrolled in this study were relatively positive about treatment and the system functioned as a tool to motivate patients to take their medications, which might have resulted in good medication adherence maintained at a high level over an 8-week period in this study.

### 4.2 Limitations and prospects

The symptoms of schizophrenia patients enrolled in this study were mild-to-moderate, and the impact of severeness of illness on medical adherence remains unclear (17). The study period was not sufficient enough to obtain data on remission and quality of life. Moreover, while the treatment of schizophrenia continues for a lifetime, it has not been determined how long it can be applicable in actual medical practice, and the practical usefulness of this system in the clinical real-world setting should be clarified. The limited number of participants restricted subgroup analysis in outcomes in this study. When using a smart phone to upload data and to review their own data, a person may feel confused when they cannot master its use or encounter mechanical problems, and some patients may feel discomfort on being monitored over 24 h by this system.

This system is also likely to be applicable not only to schizophrenia but also to other psychiatric disorders for which adherence is a concern, such as dementia, bipolar disorder, and unipolar depression, although further research is needed to confirm these findings.

## 5 Conclusion

The patch application rate was maintained throughout the study period and did not decrease after the shift to the independent phase. The proportion in which the participant appropriately applied the patch and synchronized with the application increased every week. The majority of the study participants were able to manage the series of operations early without any support. Most adverse events that occurred in the safety analysis set were classified as mild or moderate. These findings indicate that this DMS could provide a useful function in patients with schizophrenia or schizoaffective disorder in Japan. The DMS used in this study can be a support tool for monitoring medication and improving compliance objectively and accurately. It is expected that (1) healthcare professionals will be able to provide treatment based on more accurate information (2), patients will be able to be more proactive in their treatment by knowing accurate medication-related information, and (3) patients will be able to share this information with healthcare professionals, caregivers, or supporters to achieve treatment of higher quality.

## Data Availability

The data supporting the findings of this study may be accessed only with prior approval from both the corresponding author and Otsuka Pharmaceutical Co., Ltd. The data are not publicly available due to privacy or ethical restrictions.
